# Intrinsically Safe Optical Fiber Hydrogen Sensor Using Pt-SiO_2_ Coated Long-Period Fiber Grating

**DOI:** 10.3390/nano16020095

**Published:** 2026-01-12

**Authors:** Xuhui Zhang, Liang Guo, Xinran Wei, Fangzhou Mao, Yuzhang Liang, Junsheng Wang, Wei Peng

**Affiliations:** 1 Liaoning Key Laboratory of Marine Sensing and Intelligent Detection, Information Science and Technology College, Dalian Maritime University, Dalian 116026, China; xhzhang@dlmu.edu.cn (X.Z.);; 2 School of Physics, Dalian University of Technology, Dalian 116024, China

**Keywords:** optical fiber hydrogen sensor, long-period fiber grating, platinum-loaded silica nanomaterials, catalytic exothermic reaction

## Abstract

Hydrogen, a promising clean energy carrier, needs safe detection due to its flammability. Conventional electrical hydrogen sensors have drawbacks like high operating temperatures, poor selectivity and ignition risks. We propose an optical sensor using long-period fiber gratings (LPGs) coated with Pt-SiO_2_ nanomaterials. It works via catalytic reaction: H_2_ reacts with O_2_ on Pt nanoparticles, releasing heat that shifts LPG’s resonant wavelength. Structural characterization showed porous SiO_2_ with uniform Pt, ensuring efficiency and stability. Experiments proved it sensitively responds to 0.5–2.5% H_2_ (max wavelength shift 7.544 nm), with fast response/recovery, good repeatability/reversibility. Logistic fitting (R^2^ = 0.999) confirmed strong correlation. This sensor, safe, sensitive and stable, has great potential for real-time H_2_ monitoring in critical environments.

## 1. Introduction

Hydrogen, as a clean and efficient energy carrier, has experienced a significant increase in its application in recent years [1,2,3]. However, due to its high flammability and diffusivity, hydrogen can form explosive mixtures in air for volumes over 4% [4,5,6]. Consequently, hydrogen leaks during production, storage, or usage can easily lead to fire and explosion incidents, posing a major challenge to hydrogen safety monitoring [7,8,9]. These incidents have significantly impacted economic development and social stability, underscoring the critical need for highly sensitive and real-time hydrogen leakage monitoring [10,11,12,13]. Therefore, during the utilization of hydrogen energy, there is an urgent demand for a rapid, sensitive, and reliable detection method to promptly identify hydrogen leaks. An ideal hydrogen sensor should exhibit high sensitivity, excellent stability, good selectivity, and fast response time, while also offering advantages such as low cost and ease of integration [14,15,16,17].

Conventional hydrogen detection technologies primarily utilize electrical property-based sensors, such as catalytic combustion, electrochemical, and semiconductor gas sensors [18,19,20,21,22,23,24,25,26]. However, their operational mechanisms often involve electric heating or spark generation, which may pose potential ignition and explosion risks. For example, catalytic combustion sensors require heated elements to initiate hydrogen combustion, and inadequate protection may result in flame propagation. Although electrochemical sensors themselves do not intrinsically produce sparks, their operation in hydrogen-rich environments may still involve potential ignition risks associated with electrical components, such as wiring, contacts, or electronic circuitry under abnormal or fault conditions. Therefore, the deployment of such sensors in flammable and explosive environments presents a significant safety concern. Furthermore, traditional sensors are vulnerable to environmental influences: electrochemical sensors are sensitive to temperature and humidity variations and exhibit limited durability, whereas semiconductor metal oxide sensors require elevated operating temperatures and demonstrate poor selectivity due to cross-sensitivity to various flammable gases. These limitations have prompted the pursuit of safer and more reliable hydrogen detection technologies.

Optical fiber hydrogen sensors can effectively mitigate several limitations of conventional electrical sensors, particularly in terms of intrinsic safety and immunity to electromagnetic interference [27,28,29,30]. First, the optical fiber sensing head does not require electrical excitation and poses no risk of spark generation at the sensing head, thereby significantly enhancing safety in potentially explosive gas environments [31]. Second, optical fiber materials, such as quartz fibers, exhibit strong resistance to chemical corrosion [32]. Additionally, optical fibers are inherently immune to electromagnetic interference, demonstrating excellent anti-interference performance, which makes them particularly suitable for deployment in environments with strong electromagnetic noise, such as high-voltage installations and wireless communication zones [33,34]. Moreover, optical fiber sensing systems are compact and flexible, facilitating installation in confined spaces and complex wiring configurations [35]. By utilizing wavelength division multiplexing or time division multiplexing techniques, multiple sensors can be integrated along a single fiber, enabling remote monitoring and multi-point distributed detection [36,37,38,39]. This capability is especially valuable for the comprehensive monitoring of large-scale hydrogen gas distribution networks and storage tank arrays. Furthermore, optical fiber sensors offer high sensitivity and excellent resolution, allowing even minor optical changes in the fiber to be detected by high-precision demodulation systems [40,41,42]. In conclusion, optical fiber hydrogen sensors demonstrate remarkable advantages, including corrosion resistance, immunity to interference and the capability for remote real-time monitoring, thereby presenting unique technical merits in the field of hazardous gas detection [43].

Metal oxide semiconductor (MOS) sensors represent a significant class of commercially available hydrogen sensors [44]. Commonly used materials, such as SnO_2_, ZnO, and WO_3_, interact with hydrogen at specific temperatures by reacting with oxygen adsorbed on their surfaces, leading to a change in the semiconductor’s electrical resistance [45,46,47]. The advantages of MOS sensors include a simple structural design, low manufacturing cost, and sensitivity that can reach the parts-per-million (ppm) level. However, several notable limitations hinder their widespread application. First, these sensors require high-temperature operation, typically within the range of 150–450 °C, to achieve a measurable response to hydrogen [48]. This necessitates the integration of a continuous heating element, which results in high power consumption, making the sensors unsuitable for portable or battery-operated devices. Furthermore, the presence of high-temperature components poses safety risks in environments with potentially explosive gases. Second, MOS sensors suffer from inherently poor selectivity. As reducing gases, hydrogen and other flammable or reductive gases-such as carbon monoxide, methane, and ethanol can similarly reduce the surface resistance of the semiconductor. Consequently, a single MOS sensor cannot effectively distinguish hydrogen from other interfering gases, resulting in cross-sensitivity issues. Third, these sensors are highly sensitive to fluctuations in ambient humidity and temperature, which can lead to baseline drift. For instance, increased humidity levels can alter the sensor’s resistance, thereby interfering with hydrogen detection. Additionally, the requirement for high-temperature activation leads to a prolonged warm-up time before the sensor becomes operational, preventing immediate measurements upon startup. In conclusion, although MOS sensors offer high sensitivity and are based on well-established technology, their drawbacks-including high operational temperature, significant power consumption, limited selectivity, and susceptibility to environmental factors-impose constraints on their use in hydrogen detection, particularly in safety-critical monitoring applications.

In response to the limitations of conventional hydrogen sensors, a solution is offered by fiber optic hydrogen sensors based on long-period fiber gratings (LPG) coated with platinum-loaded silica (Pt-SiO_2_) nanomaterials. This study demonstrates a thermally transduced hydrogen sensing mechanism that combines a porous Pt-SiO_2_ catalytic layer with the high temperature sensitivity of an LPG, enabling intrinsically safe and reversible hydrogen detection. First, this sensor operates entirely on optical principles, eliminating the need for electrical power or electronic components at the sensing location. This design inherently prevents the risk of electrical sparking, thereby ensuring intrinsic safety in environments where hydrogen is present. The fiber optic sensing head can be located hundreds of meters or even kilometers away from the light source and detector, connected solely by optical fibers, enabling remote and off-site monitoring. In the event of a hydrogen leak, personnel can obtain measurement signals from a safe distance without entering hazardous zones. This isolation design significantly enhances both the safety and operational flexibility of hydrogen detection in high-risk environments. Second, the LPG-Pt-SiO_2_ sensor overcomes durability issues commonly associated with traditional sensing elements by utilizing robust optical and catalytic materials. Platinum, as a catalytic material, is a noble metal that remains chemically stable and is not consumed during the reaction process. Furthermore, the SiO_2_ support typically features a porous structure, which provides resistance to powdering and poisoning, thereby maintaining long-term catalytic performance. These characteristics demonstrate that the fiber optic sensor offers reliability and environmental adaptability over extended periods of operation.

More importantly, the LPG-Pt-SiO_2_ sensor employs a catalytic exothermic detection mechanism. When hydrogen interacts with the Pt catalyst, it does not induce lattice phase transformations as seen in palladium, but instead directly catalyzes the reaction between hydrogen and oxygen, generating heat. The reaction ceases immediately upon removal of hydrogen, avoiding the hysteresis associated with phase changes [49]. This thermal response is both instantaneous and reversible. Any change in hydrogen concentration leads to a rapid adjustment in the reaction rate and heat output, resulting in fast sensor response and recovery times without significant delay. Additionally, since the sensing signal is derived from thermal changes rather than structural modifications, the sensor shows no observable memory effect within the tested hydrogen concentration range and cycling conditions. In conclusion, the LPG-Pt-SiO_2_ fiber optic hydrogen sensor achieves operational safety through passive optical measurement, longevity through material stability, and enhanced performance in terms of speed and selectivity through catalytic thermal effects, effectively addressing many of the limitations of traditional Pd-based, MOS, and Micro-Electro-Mechanical System (MEMS) hydrogen sensors.

## 2. Materials and Methods

A long-period fiber grating is a passive optical device characterized by a periodic modulation of the refractive index within the core of an optical fiber [50,51]. Its primary function is to enable the coupling of energy from the fundamental mode propagating through the fiber core to a specific order of cladding mode traveling in the same direction. This coupling can occur most efficiently at certain wavelengths that satisfy the phase-matching condition, which are known as resonant wavelengths (λ*_res_*). In the transmission spectrum, one or more attenuation peaks-manifested as spectral minima-appear at these resonant wavelengths due to energy coupling losses. The phase-matching condition is mathematically expressed by the following equation:

(1) $$\lambda_{res}=(n_{eff,co}-n_{eff,cl}^{\left(m\right)})\cdot \Lambda$$

λ*_res_* is the resonant wavelength, that is, the wavelength corresponding to the minimum value observed in the spectrum. *n_eff_*_,*co*_ represents the effective refractive index of the fundamental mode within the core. *n_eff_*_,*cl*_ represents the effective refractive index of the m-th order cladding mode. Λ refers to the grating period.

The variation of temperature will affect two key parameters in the phase-matching condition formula: the difference in effective refractive index and the grating period. We can analyze the drift of the resonant wavelength with temperature by taking the total differential of the phase-matching formula with respect to temperature (T):

(2) $$S_{T}=\frac{d\lambda_{res}}{dT}=\frac{d}{dT}[(n_{eff,co}-n_{eff,cl}^{\left(m\right)})\cdot \Lambda ]=\Lambda \cdot \frac{d(n_{eff,co}-n_{eff,cl}^{\left(m\right)})}{dT}+(n_{eff,co}-n_{eff,cl}^{\left(m\right)})\cdot \frac{d\Lambda }{dT}$$

This formula clearly demonstrates two independent physical effects: the first term of the formula describes the thermo-optic effect. The refractive index of the material changes with temperature, and this phenomenon is called the thermo-optic effect. The core (germanium-doped quartz) and cladding (pure quartz) of the optical fiber have different thermo-optic coefficients. The thermo-optic coefficient of the germanium-doped core is slightly greater than that of the pure quartz cladding, meaning that when the temperature rises, the refractive index of the core increases faster than that of the cladding. Therefore, the effective refractive index of the core mode and the effective refractive index of the cladding mode both increase with temperature, but at different rates. Eventually, this leads to a change in the effective refractive index difference with temperature. For most standard optical fibers, when the temperature rises, the resonant wavelength *λ_res_* shifts towards longer wavelengths. This is the main cause of wavelength drift. The second term of the formula describes the thermal expansion effect. The physical dimensions of materials will elongate or contract with changes in temperature. The optical fiber itself will undergo thermal expansion due to an increase in temperature, causing the physical period Λ of the grating to lengthen. This length change can be described by the coefficient of thermal expansion. For quartz optical fibers, the coefficient of thermal expansion is a very small positive value. Therefore, the contribution of the second term is also positive. It will also cause the resonant wavelength λ*_res_* to shift towards longer wavelengths when the temperature rises. For a standard quartz fiber LPG, the contribution of the thermo-optic effect is much greater than that of the thermal expansion effect. Since the contributions of both effects are positive, their sum must be positive. Therefore, the total temperature sensitivity is a positive value. This perfectly explains the phenomenon observed in the experiment: when the temperature rises (*dT* > 0), the resonant wavelength shifts to a longer wavelength (*d*λ*_res_* > 0), that is, a red shift occurs. When the temperature drops (*dT* < 0), the resonant wavelength shifts to a shorter wavelength (*d*λ*_res_* < 0), that is, a blue shift occurs.

**Figure 1:**
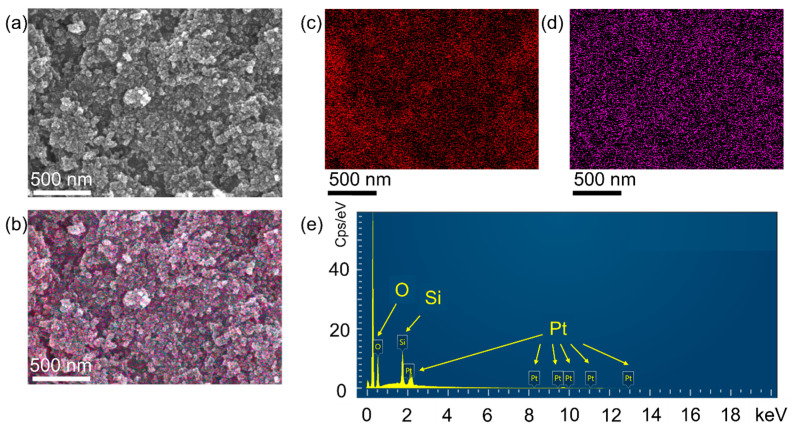
The microstructure and elemental distribution of platinum-loaded silica (Pt/SiO_2_) nanocomposites. (**a**) The secondary electron image of the material obtained by scanning electron microscopy (SEM). (**b**) The distribution maps of platinum (purple) and silicon (red). (**c**) Elemental mapping images of Si. (**d**) Elemental mapping images of Pt. (**e**) Energy dispersive spectrum (EDS).

This series of Figure 1 illustrates the microstructure and elemental distribution of platinum-loaded silica (Pt-SiO_2_) nanocomposites. Figure 1a shows the secondary electron image of the material obtained by scanning electron microscopy (SEM), presenting its surface morphology. It can be observed from the figure that the material is composed of many nanoscale particles that aggregate to form a porous agglomerate structure. The particle sizes are relatively uniform, and some brighter particles can be seen on the surface, which usually indicates that the area is composed of elements with higher atomic numbers. It is preliminarily speculated that these are platinum (Pt) nanoparticles. Figure 1b superimposes the distribution maps of platinum (purple) and silicon (red). Through the superimposed image, it can be more intuitively seen that the platinum nanoparticles are closely combined with the silica support and are distributed very uniformly, without obvious agglomeration. This indicates that platinum is well dispersed in the composite material. Figure 1c,d are elemental mapping images. In them, the red parts represent the distribution of silicon, and the purple parts represent the distribution of platinum. Silicon, as the base material, is uniformly distributed throughout the field of view, forming the framework of the material. While platinum is highly dispersed in the form of small purple spots on the silicon base. Figure 1e is the energy dispersive spectrum (EDS) of this area. The spectrum clearly shows the characteristic energy peaks of oxygen (O), silicon (Si), and platinum (Pt). The presence of oxygen and silicon peaks confirms that the base material is silica (SiO_2_). At the same time, the appearance of multiple characteristic peaks of platinum conclusively proves the existence of platinum. The relative intensity of the spectral peaks also indicates that the material is mainly composed of silica, with platinum as the minor component loaded on it.

**Figure 2:**
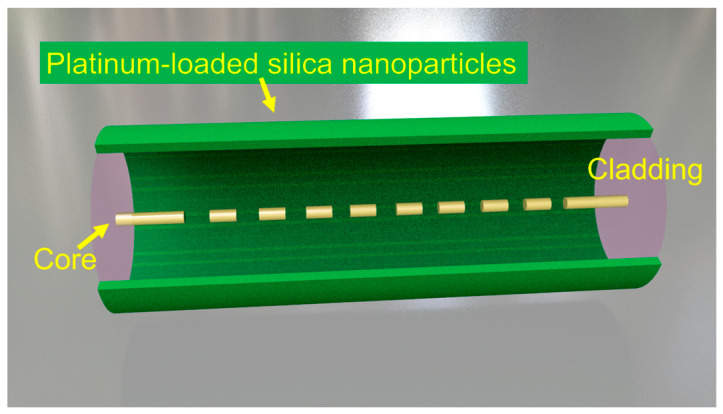
Hydrogen-induced exothermic optical fiber hydrogen sensor based on platinum-loaded silica nanoparticles and long-period fiber grating.

Figure 2 shows a hydrogen-induced exothermic optical fiber hydrogen sensor based on platinum-loaded silica nanoparticles and long-period fiber grating (LPG). This sensor ingeniously combines the chemical properties of nano-catalytic materials with the optical characteristics of fiber gratings, forming a highly sensitive and intrinsically safe optical hydrogen detection device. The sensing substrate is a long-period fiber grating, LPG, which is a periodic refractive index modulation structure formed in the core of a standard single-mode fiber through ultraviolet laser exposure technology. Its “long period” refers to the fact that its grating period is much longer than that of a fiber Bragg grating (FBG). The function of LPG is to achieve the coupling of guided modes in the fiber. It couples the light energy of the forward core mod propagating in the core to the specific order of cladding modes propagating in the same direction. This coupling effect is manifested in the transmission spectrum as a series of loss peaks. The wavelength corresponding to each resonant peak (λ*_res_*) is highly sensitive to the parameters of the external environment, especially temperature and environmentally refractive index. In this hydrogen sensor, LPG mainly utilizes its high sensitivity to temperature. It serves as a high precision “optical fiber thermometer” and is the transducer element of the entire sensor, responsible for converting the heat changes generated by the chemical reaction into measurable spectral signals. Pt-SiO_2_ nanoparticles are the core recognition and reaction part of the sensor, coated in the form of a thin film on the area where the LPG is located. SiO_2_ nanoparticles have a high specific surface area and a porous network structure of nanoparticles. This porous structure provides channels for the diffusion of hydrogen and oxygen and provides a large attachment area for platinum nanoparticles. It provides a highly dispersed and stable carrier for platinum catalysts, preventing the agglomeration of platinum nanoparticles. SiO_2_ material can adhere well to the quartz surface of the optical fiber, ensuring the firmness of the sensitive film. Its porosity allows hydrogen and oxygen in the environment to quickly penetrate the interior of the film and meet platinum particles. Pt nanoparticles are highly dispersed platinum metal particles with a size typically ranging from a few nanometers to tens of nanometers, uniformly embedded or loaded in the silica nano-matrix. Pt is an efficient catalyst for hydrogen oxidation. When hydrogen molecules encounter the surface of platinum nanoparticles, they are catalyzed at room temperature or slightly higher temperatures and undergo a vigorous oxidation-reduction reaction with oxygen in the environment. The structure of the entire sensor is to uniformly coat the platinum-loaded silica nanoparticles on the external cladding surface of the already fabricated long-period fiber grating, forming a layer of controllable thickness and porous composite sensitive film.

**Figure 3:**
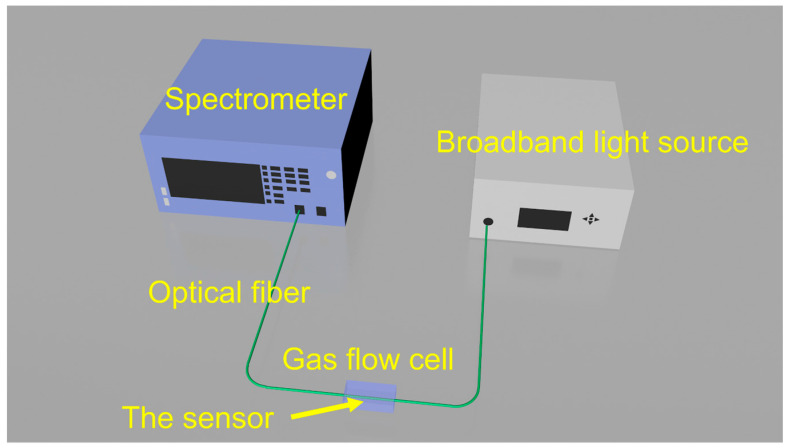
The hydrogen-induced exothermic fiber optic hydrogen sensing system.

As shown in Figure 3, the hydrogen-induced exothermic fiber optic hydrogen sensing system based on long-period fiber gratings utilizes the exothermic effect produced when a catalyst meets hydrogen to convert the chemical signal of hydrogen concentration in the environment into a temperature signal. This temperature signal is then transformed into a measurable spectral signal by a long-period fiber grating that is sensitive to temperature. The system consists of the following components. A 1550 nm band broadband light source. The sensing principle of the long-period fiber grating is based on one or more “resonant absorption peaks” in its transmission spectrum. These absorption peaks will undergo wavelength drift with temperature changes. The broadband light source emits light covering the entire possible wavelength range of drift, ensuring that the spectrometer can capture the peak completely regardless of its movement. The sensor is the core sensing element of the system, responsible for converting changes in hydrogen concentration in the environment into changes in the fiber optic spectral signal through physical and chemical effects. A long-period fiber grating is a periodically modulated refractive index structure formed within the core of an optical fiber. Its function is to couple the light transmitted in the core to the cladding at specific wavelengths when broadband light passes through, thereby forming a loss peak in the transmission spectrum. To enable the long-period fiber grating, which is not directly sensitive to hydrogen, to detect hydrogen, a sensitive material that catalyzes hydrogen must be coated on the external area of the grating. The gas flow cell provides a sealed and controllable measurement environment, ensuring that the sensor can react fully and stably with the gas to be measured. The flow cell is designed with an inlet and an outlet, allowing the gas to be measured to flow in and react with the sensor, and then be discharged. This ensures the real-time nature of the measurement. The spectrometer can precisely measure and analyze the light signal after passing through the sensor and track the position and change of the resonant wavelength in real time. When hydrogen enters the gas flow cell and the sensor temperature rises, causing the resonant peak to drift, the spectrometer can capture the wavelength change with high resolution.

In an environment without hydrogen, the light emitted by a broadband light source passes through the LPG, and the spectrometer measures a stable transmission spectrum and records the initial resonant wavelength *λ_initial_*. When the sensor is exposed to an environment containing hydrogen, hydrogen (H_2_) and oxygen (O_2_) molecules in the air diffuse through the porous SiO_2_ film and reach the surface of the internal Pt nanoparticles. Under the catalytic action of Pt, H_2_ and O_2_ undergo a vigorous oxidation reaction, generating water and releasing a large amount of heat (exothermic reaction). The generated heat is rapidly transferred to the closely contacting LPG, causing a significant increase in the local temperature of the fiber segment where the LPG is located. Due to the high sensitivity of the LPG’s resonant wavelength to temperature, the increase in temperature causes a drift in its resonant wavelength. The spectrometer detects a new resonant wavelength λ*_final_*. The drift of the wavelength is directly proportional to the temperature increase of the LPG, and the temperature increase is directly related to the rate of the hydrogen catalytic reaction, which ultimately depends on the concentration of hydrogen in the environment. Therefore, by monitoring the drift of the resonant wavelength, the hydrogen concentration in the environment can be accurately deduced.

## 3. Results and Discussion

**Figure 4:**
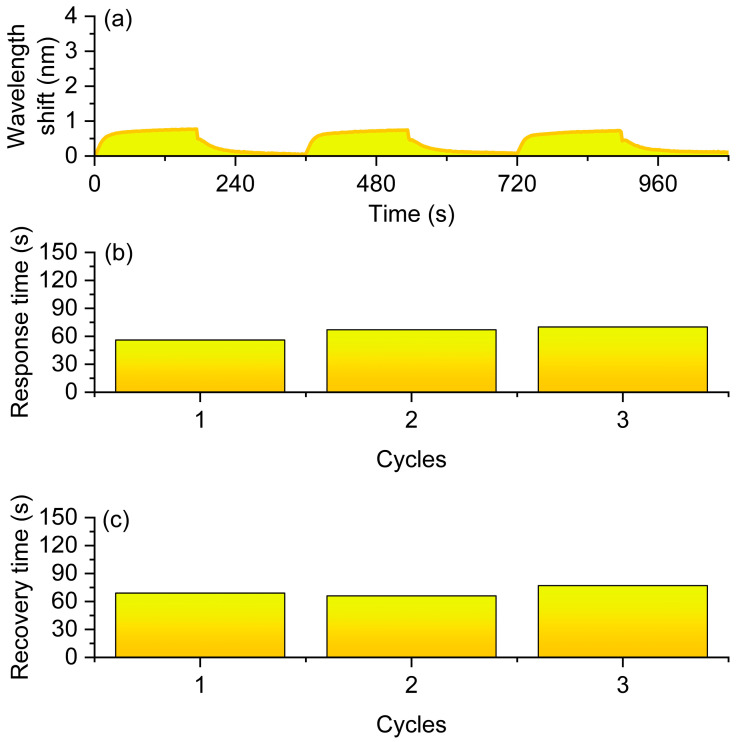
Sensing performance of the optical fiber hydrogen sensor in 0.5% H_2_ atmosphere. (**a**) Resonance wavelength shift curves during three H_2_–air cycles. (**b**) Response times of the three cycles. (**c**) Recovery times of the three cycles.

To evaluate the gas sensing performance of the sensor, it was mounted within a sealed gas testing chamber. The sensor operates under ambient conditions without external heating. During the experimental process, the gas flow rate was precisely regulated using a mass flow controller to ensure the stability of atmospheric composition and concentration. In this work, the response time is defined as the time required for the wavelength to reach 90% of its total shift after hydrogen exposure, while the recovery time is defined as the time required for the wavelength to return to within 10% of its initial baseline after hydrogen removal. The hydrogen volume fraction was maintained at 0.5%. Each measurement cycle consisted of a 3-min exposure to hydrogen gas followed by a 3-min air purging phase, with a total of three consecutive cycles conducted. Throughout the experiment, a spectrometer continuously monitored and recorded the transmission spectrum of the long-period fiber grating in real time, enabling the observation of resonance wavelength shifts over time. The experimental results are summarized in Figure 4. Figure 4a illustrates the resonance wavelength shift curves across the three cycles, showing a clear redshift during hydrogen exposure and partial recovery during the air purging phase. These observations indicate that the sensor exhibits a rapid and reversible spectral response to low-concentration hydrogen. Figure 4a compares the hydrogen-induced resonance wavelength shifts among the three cycles, with an average shift of 0.703 nm, demonstrating that the sensor maintains high sensitivity and good repeatability across multiple cycles. Figure 4b,c depict the response time and recovery time, respectively, with average values of 64 s and 71 s, both remaining within approximately one minute. This indicates that the sensor possesses a fast response speed and favorable reversibility during hydrogen detection. The integration of platinum-doped silica nanoparticles with long-period fiber gratings significantly enhances the sensor’s sensitivity and response speed under low-concentration hydrogen conditions, while maintaining stable gas sensing performance over repeated cycles.

**Figure 5:**
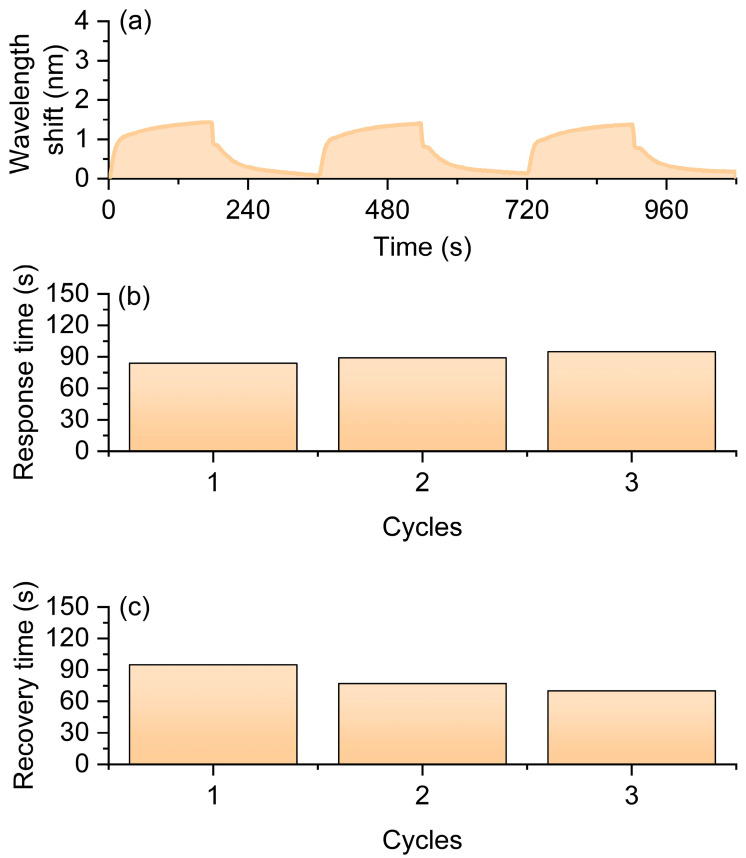
Optical fiber hydrogen sensor based on Pt-loaded SiO_2_ nanomaterials tested under 1% H_2_. (**a**) Resonant wavelength shift curves over three cycles with alternating H_2_ and air exposure. (**b**) Response times of the three cycles. (**c**) Recovery times of the three cycles.

Further cyclic tests were conducted in a 1% hydrogen environment. The experimental conditions were set to alternate hydrogen and air for 3 min each, with a total of three cycles. The results are shown in Figure 5. The spectral response curve in Figure 5a indicates that the sensor consistently experiences a stable redshift in resonance wavelength under the influence of hydrogen and gradually returns to its initial state in the air environment. Further analysis reveals that the average maximum wavelength shift in the three cycles is 1.337 nm (Figure 5a), which is close and remains at a relatively high level, demonstrating the sensor’s good stability and repeatability in repeated detections. In terms of dynamic characteristics, the average response time for the three cycles is 89 s (Figure 5b), and the average recovery time is 81 s (Figure 5c). The results show that the sensor can complete the response and recovery within the order of hundreds of seconds, indicating a relatively fast dynamic characteristic.

**Figure 6:**
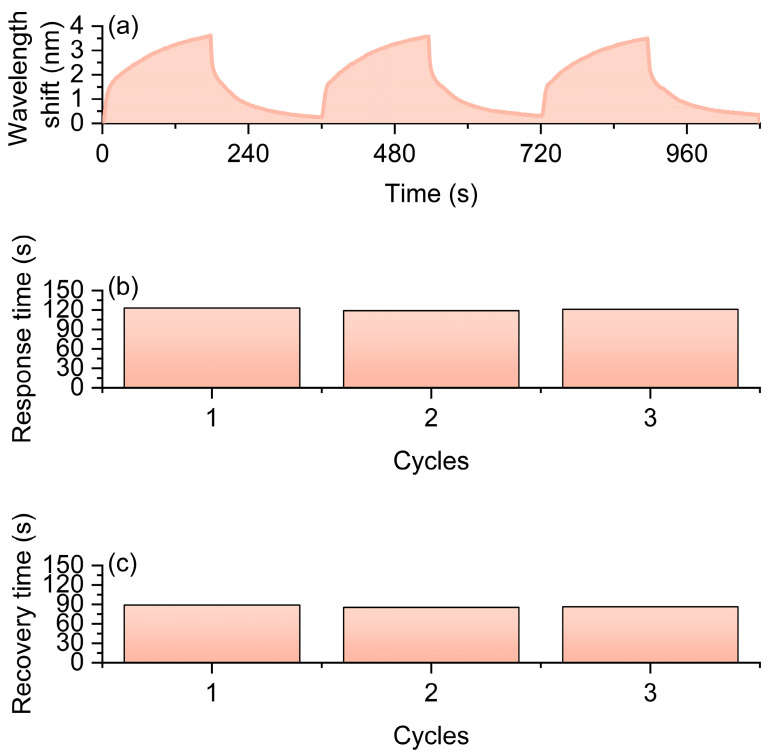
Experimental results of the optical fiber hydrogen sensor under 1.5% H_2_ concentration. (**a**) Resonance wavelength shift curves during three successive H_2_/air cycles. (**b**) Response times of the sensor in three cycles. (**c**) Recovery times of the sensor in three cycles.

Under a hydrogen concentration of 1.5%, the sensor was subjected to three hydrogen/air alternating cycle experiments, with each cycle involving a 3-min hydrogen and air exposure time. The experimental results are shown in Figure 6: Figure 6a presents the resonance wavelength variation curves of the sensor over time during the three cycles, all demonstrating a distinct hydrogen-induced redshift and its reversible process. Specifically, the average wavelength shift across the three cycles was 3.376 nm (Figure 6a), indicating a stable and considerable spectral response of the sensor under repeated cycling. The average response time was 121 s (Figure 6b), suggesting its ability to complete the sensing process rapidly at higher hydrogen concentrations. Meanwhile, the average recovery time was 87 s (Figure 6c), showing a relatively consistent desorption behavior. The sensor maintained good response repeatability and stability throughout multiple cycles, and the significant wavelength shift indicates its high sensitivity. Despite minor fluctuations in different cycles, the overall trend suggests that this sensor exhibits excellent reversibility and reliability in high-concentration hydrogen detection.

**Figure 7:**
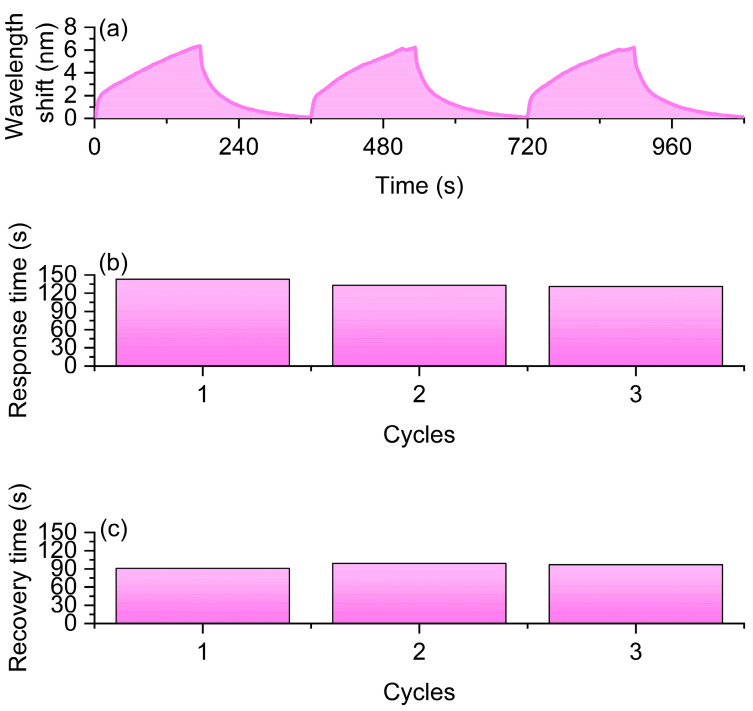
Experimental results of the fiber-optic hydrogen sensor under 2% hydrogen concentration. (**a**) Resonance wavelength shift curves during three hydrogen exposure/recovery cycles. (**b**) Response times measured in three cycles under 2% hydrogen. (**c**) Recovery times measured in three cycles after hydrogen removal.

Under a hydrogen concentration of 2%, the sensor was tested three times in a cycle experiment where hydrogen and air were introduced for 3 min each, as shown in Figure 7. As depicted in Figure 7a, during the three cycles of hydrogen introduction and evacuation, the resonance wavelength experienced significant redshifts. The average shift in resonance wavelength caused by hydrogen was 6.193 nm (see Figure 7a), with results relatively consistent and the maximum deviation less than 5%, demonstrating the sensor’s good stability in repeated experiments. The large wavelength drift further indicates that the loaded platinum nanomaterials can effectively promote hydrogen adsorption and exothermic reactions, thereby causing significant changes in the refractive index around the optical fiber. In terms of dynamic response characteristics, the average response time of the sensor was 136 s (Figure 7b), while the average recovery time was 96 s (Figure 7c). The distribution of response and recovery times was relatively concentrated, suggesting that the sensor has good reversibility and cycling stability during hydrogen adsorption and desorption, and the kinetic process is relatively balanced. The optical fiber hydrogen sensor not only exhibited a large resonance wavelength drift at a 2% hydrogen concentration but also demonstrated stable and reliable response and recovery characteristics.

**Figure 8:**
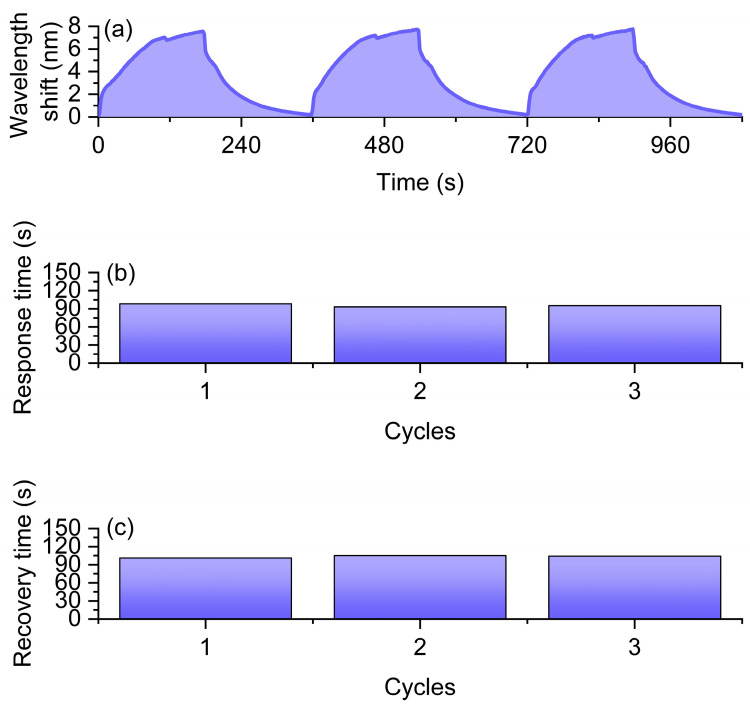
Experimental results of the fiber-optic hydrogen sensor under 2.5% H_2_ atmosphere. (**a**) Resonant wavelength shift curves over three successive cycles with alternating hydrogen and air exposure. (**b**) Response times of the three cycles. (**c**) Recovery times of the three cycles.

As shown in Figure 8, the device was continuously operated three times under 2.5% H_2_ conditions with a “hydrogen-air” cycle of 3 min each. Figure 8a shows that the time-domain curve rapidly rises and reaches a stable plateau after each hydrogen exposure and returns to the initial baseline during air exposure, demonstrating excellent reversibility and baseline stability. Correspondingly, the hydrogen-induced resonance wavelength shifts in Figure 8a are highly consistent, with an average of 7.544 nm, indicating excellent repeatability of the device output at this concentration. From the statistics in Figure 8b,c, the average response time is 95 s and the average recovery time is 103 s, with values close to each other, suggesting that the kinetics of the device during hydrogenation and dehydrogenation are relatively symmetrical and the cycle efficiency is high. These behaviors can be attributed to the catalytic exothermic reaction of H_2_ on the Pt surface, causing local temperature rise, which changes the effective refractive index of the LPG cladding mode and leads to redshift of the resonance wavelength; as air replaces the hydrogen and the temperature drops, the wavelength returns to its initial value. Overall, this sensor achieves significant spectral shifts, fast response/recovery rates, and good cycle consistency under percentage-level hydrogen, demonstrating its potential for application in flammable gas safety monitoring and process control.

**Figure 9:**
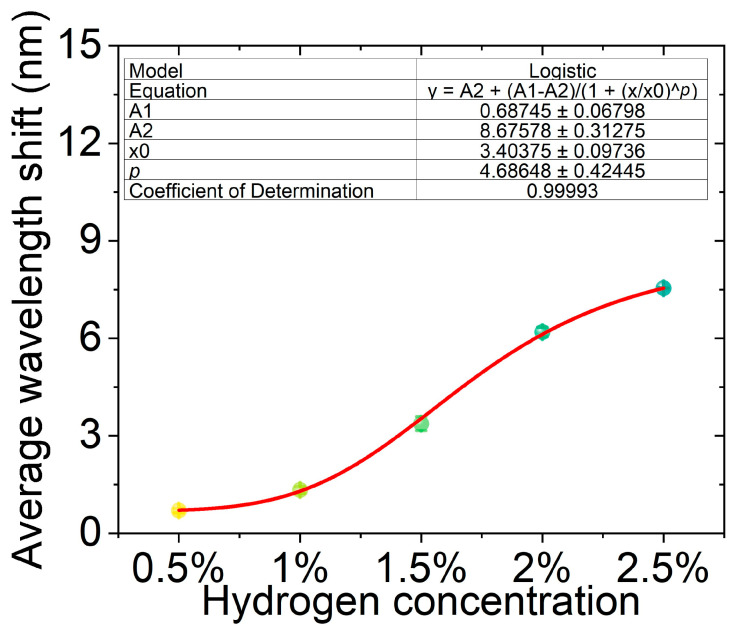
Average wavelength shift of the hydrogen sensor at different hydrogen concentrations (0.5–2.5%). Error bars indicate standard deviations, and the red curve represents the logistic fit (R^2^ = 0.999).

Based on the above hydrogen concentration cycling test results, the relationship curve between the average wavelength response and hydrogen concentration was plotted, as shown in Figure 9. With the increase of hydrogen concentration, the hydrogen-induced resonance wavelength shift shows a significant growth trend, and the corresponding standard deviations are relatively small (within the range of 0.065–0.215 nm), indicating that the experimental data have good stability and repeatability. To better reveal the relationship between wavelength shift and hydrogen concentration, the experimental data were fitted with a logistic function, and the resulting coefficient of determination was as high as 0.999, indicating that this model can accurately describe the nonlinear relationship between hydrogen concentration and optical response. The overall results show that this fiber optic hydrogen sensor exhibits sensitive response characteristics at low concentrations and tends to saturate in the high concentration region, conforming to the typical adsorption-reaction kinetics law, which verifies its potential as a high-performance hydrogen detector.

To interpret the nonlinear dependence of the wavelength shift on hydrogen concentration, the experimental data were fitted using a logistic function. This choice is not purely mathematical but is physically motivated by the catalytic reaction kinetics of hydrogen on Pt nanoparticles. At low hydrogen concentrations, the reaction rate is governed by hydrogen adsorption on available Pt active sites, resulting in an approximately linear increase in released heat and wavelength shift. As the hydrogen concentration increases, the number of available catalytic sites gradually becomes saturated, leading to a reduced incremental heat generation rate and saturation behavior in the optical response. The logistic model therefore serves as an effective phenomenological representation of this transition from a linear regime to a saturation regime.

At lower hydrogen concentrations, partial baseline recovery is observed during the air purging stage. This partial recovery does not indicate irreversible material degradation but reflects the thermal inertia of the catalytic coating. In contrast, full baseline recovery is achieved at higher hydrogen concentrations, where sufficient heat exchange accelerates temperature equilibration. The increase in response and recovery times with hydrogen concentration can be explained by the enhanced catalytic reaction rate at higher hydrogen partial pressures, which generates greater thermal energy and requires longer equilibration times. The relatively balanced response and recovery kinetics indicate stable adsorption–reaction–desorption processes on the Pt surface.

In the Supplementary Materials, Figure S1 shows the repeatability of the response time and recovery time. Figure S1a,b presents the average response time and recovery time of the sensor at different hydrogen concentrations (0.5–2.5%), with error bars representing the standard deviations calculated from three repeated hydrogen/air cycles at each concentration. The small error bars and consistent trends across all hydrogen concentrations confirm that both response time and recovery time possess good statistical repeatability.

To directly verify the actual operating temperature of the sensing element during hydrogen exposure, we performed independent infrared (IR) thermal measurements under a representative hydrogen concentration of 2.5% H_2_, which corresponds to the highest response regime discussed in the paper. The IR measurements were carried out using a calibrated infrared camera (PC200, Guide sensmart, Wuhan, China). The results show that during hydrogen exposure, the maximum surface temperature of the Pt-SiO_2_-coated sensing region reached approximately 88.1 °C, while the surrounding environment remained close to room temperature, as shown in the Supplementary Materials, Figure S2. These data confirm that the temperature rise originates exclusively from the hydrogen-induced catalytic exothermic reaction on Pt, rather than from external heating. The sensing mechanism in our work relies on Pt-catalyzed hydrogen oxidation, which proceeds efficiently at room temperature and above, generating immediate local heating without involving structural phase changes. As a result, the sensor exhibits a fast and reversible response even under ambient conditions.

**Figure 10:**
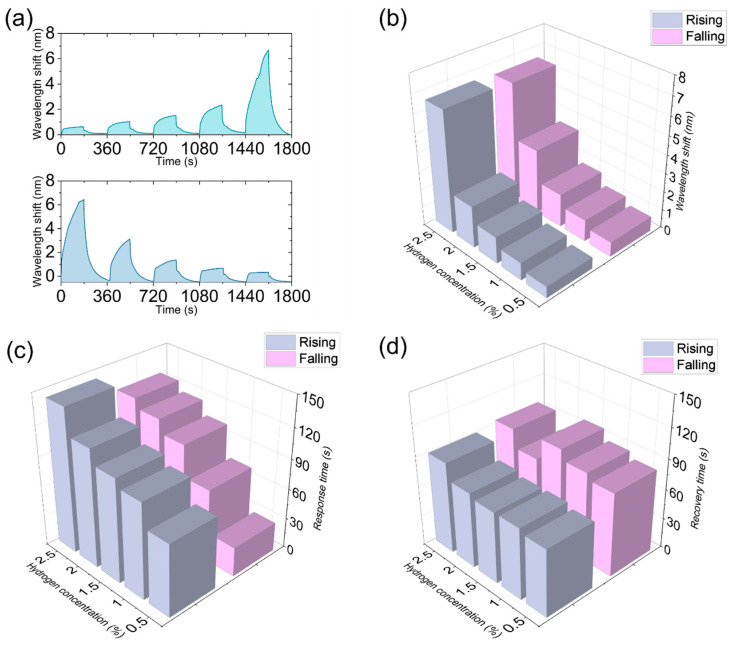
Hydrogen sensing performance of the optical fiber sensor under continuously varying concentrations. (**a**) Resonance wavelength shift response curves during concentration increase and decrease. (**b**) Resonance wavelength shift amplitudes at different concentrations. (**c**) Response times. (**d**) Recovery times.

Furthermore, we conducted a hydrogen detection experiment with continuously varying concentrations. The response of the optical fiber hydrogen sensor to different concentrations of hydrogen showed a clear concentration dependence, as illustrated in Figure 10. As shown in the upper graph of Figure 10a, when the hydrogen concentration increased successively (0.5%, 1.0%, 1.5%, 2.0%, 2.5%), the resonance wavelength shifts gradually increased, and the response amplitude significantly enhanced at high concentrations, indicating that the sensor has good sensitivity and resolution to hydrogen concentration changes. Conversely, in the lower graph of Figure 10a, when the hydrogen concentration decreased successively, the wavelength shifts gradually decreased, and the recovery curve indicated that the sensor could gradually return to its initial state, demonstrating its good reversibility and stability within different concentration ranges. Notably, there were certain differences in the wavelength response amplitude and kinetic characteristics during the concentration increase and decrease processes, which might be related to the rate differences in the adsorption and desorption processes of hydrogen in the sensitive layer. Overall, these experimental results verified that the designed optical fiber hydrogen sensor has sensitive, reversible, and stable response characteristics within a wide concentration range. As shown in the data of Figure 10b, as the hydrogen concentration increased from 0.5% to 2.5%, the resonance wavelength shift of the sensor continuously increased. During the concentration increase stage, the wavelength shift increased from 0.612 nm to 6.56 nm, showing a clear enhancement trend; during the concentration decrease stage, the corresponding wavelength shift increased from 0.789 nm to 6.455 nm, also demonstrating a gradually increasing trend with the increase in concentration. Overall, the response trends in the increase and decrease stages were highly consistent, indicating that the sensor has good repeatability and reversibility during the concentration cycling process and can maintain a stable sensitive response under different concentration conditions. As shown in Figure 10c, from the response time data, as the hydrogen concentration increased from 0.5% to 2.5%, the response time of the sensor in the increase stage increased from 72 s to 143 s, showing a gradually extending trend; in the decrease stage, the response time increased from 29 s to 125 s, also increasing with the increase in concentration. Overall, the response trends in the two stages were consistent and showed good regularity within different concentration ranges. In the low concentration range (0.5–1.0%), the response time in the decrease stage was shorter, demonstrating the sensor’s rapid response to hydrogen concentration changes; while at higher concentrations, the response times in the increase and decrease stages gradually approached, reflecting the consistency of the material during the adsorption and desorption processes. As shown in Figure 10d, from the recovery time data, as the hydrogen concentration increased from 0.5% to 2.5%, the recovery time of the sensor in the increase stage increased from 67 s to 91 s, showing a gradually extending trend; in the decrease stage, the recovery time varied within the range of 77–99 s, with an overall trend similar to that in the increase stage. Overall, the recovery curves in the two stages maintained good consistency, indicating that the sensor could stably return to its initial state during both adsorption and desorption processes. Overall, these results demonstrated that the sensor had stable recovery performance and good repeatability during the hydrogen concentration increase and decrease processes, providing a reliable basis for its application in dynamic hydrogen monitoring.

Compared with related studies, this research has certain advantages. The response time of hydrogen-induced exothermic sensors based on thermistors can reach two minutes [52]. In contrast, this sensor detects hydrogen by monitoring the wavelength drift of the optical resonance of long-period fiber gratings, which responds rapidly and can provide earlier warnings of hydrogen leakage. Within the hydrogen concentration range of 0.5% to 2.5% by volume, this sensor generates significant wavelength drift, allowing for easy identification of subtle changes in hydrogen concentration within this range. In contrast, some highly sensitive fiber optic sensors, although having a lower detection limit, have a measurement range limited to less than 1% hydrogen by volume [53]. This sensor’s detection range extends up to 2.5%, providing a clear signal at low concentrations around 0.5%, offering a wider practical application range. The sensor exclusively uses optical signal transmission and detection, with the sensing head having no risk of electrical sparks, thus being intrinsically safe in flammable gas environments. Traditional electrochemical or semiconductor sensors have powered components and may even require heated elements during operation, potentially causing electrical sparks and posing dangers [54]. Fiber optic sensors, however, do not require electrical excitation and are particularly suitable for use in flammable and explosive environments such as hydrogen. This advantage is crucial in hydrogen safety monitoring.

## 4. Conclusions

This work demonstrates the design and performance of a hydrogen sensor based on long-period fiber gratings coated with platinum-loaded silica nanomaterials. By employing an optical measurement mechanism, the sensor eliminates the ignition risks associated with conventional electrical sensors, ensuring intrinsic safety. The Pt-SiO_2_ sensitive layer provides a porous structure with highly dispersed Pt nanoparticles, enabling efficient catalytic reactions with hydrogen and maintaining long-term stability. Experimental investigations confirmed that the sensor exhibits high sensitivity, rapid response and recovery, excellent reversibility, and stable repeatability over multiple hydrogen exposure cycles. Moreover, the resonance wavelength response shows a strong nonlinear correlation with hydrogen concentration, accurately described by a logistic model. In terms of sensing performance, the sensor exhibits a practically wide hydrogen detection range from 0.5 to 2.5%, covering the concentration region most relevant to early hydrogen leakage warning and safety monitoring. Within this range, the sensor produces large and clearly resolvable resonance wavelength shifts, reaching up to 7.544 nm at 2.5% H_2_, while still providing a distinct response of approximately 0.7 nm at 0.5% H_2_. Such signal amplitudes enable reliable discrimination of low-concentration hydrogen without requiring ultra-high-resolution demodulation systems. These results highlight the advantages of the proposed sensor in terms of safety, sensitivity, and durability, demonstrating its potential as a reliable solution for hydrogen leakage detection and dynamic monitoring in hydrogen energy applications.

## Data Availability

The original contributions presented in this study are included in the article. Further inquiries can be directed to the corresponding author.

## Supplementary Materials

The following supporting information can be downloaded at: https://www.mdpi.com/article/10.3390/nano16020095/s1. Figure S1. The repeatability of the response time and recovery time. (a) and (b) present the average response time and recovery time of the sensor at different hydrogen concentrations (0.5–2.5%); Figure S2. The infrared measurements.

## References

[1] Zhao H., Yanqi X. (2025). Risk assessment of zero-carbon hydrogen energy storage systems coupled with renewable energy power generation systems. Int. J. Hydrogen Energy.

[2] Grabowska-Musiał E., Drzeżdżon J., Jacewicz D. (2025). Advances and challenges in hydrogen generation using metal complex-semiconductor hybrid systems for sustainable energy applications. Int. J. Hydrogen Energy.

[3] Evro S., Jain I.P. (2025). MXenes for hydrogen energy systems: Advances in production, storage, fuel cells, and safety applications. Int. J. Hydrogen Energy.

[4] Sangchap M., Hashtroudi H., Thathsara T., Harrison C.J., Kingshott P., Kandjani A.E., Trinchi A., Shafiei M. (2024). Exploring the promise of one-dimensional nanostructures: A review of hydrogen gas sensors. Int. J. Hydrogen Energy.

[5] Ping X., Cao X., Cao C., Lei H., Yang C., Cheng Q., Zhou T., Liu M. (2024). Fiber Grating Hydrogen Sensor: Progress, Challenge and Prospect. Adv. Sens. Res..

[6] Sahoo T., Kale P. (2021). Work Function-Based Metal–Oxide–Semiconductor Hydrogen Sensor and Its Functionality: A Review. Adv. Mater. Interfaces.

[7] Darmadi I., Nugroho F.A.A., Langhammer C. (2020). High-Performance Nanostructured Palladium-Based Hydrogen Sensors—Current Limitations and Strategies for Their Mitigation. ACS Sens..

[8] Li Y., Wang Z., Shi X., Fan R. (2023). Safety analysis of hydrogen leakage accident with a mobile hydrogen refueling station. Process Saf. Environ. Prot..

[9] Huan N., Yamamoto T., Sato H., Tzioutzios D., Yin H., Sala R. (2024). Does accident awareness affect people’s risk perception of hydrogen infrastructure and information-seeking behaviour?. Appl. Energy.

[10] Darmadi I., Nugroho F.A.A., Kadkhodazadeh S., Wagner J.B., Langhammer C. (2019). Rationally Designed PdAuCu Ternary Alloy Nanoparticles for Intrinsically Deactivation-Resistant Ultrafast Plasmonic Hydrogen Sensing. ACS Sens..

[11] Martvall V., Klein Moberg H., Theodoridis A., Tomeček D., Ekborg-Tanner P., Nilsson S., Volpe G., Erhart P., Langhammer C. (2025). Accelerating Plasmonic Hydrogen Sensors for Inert Gas Environments by Transformer-Based Deep Learning. ACS Sens..

[12] Nugroho F.A.A., Bai P., Darmadi I., Castellanos G.W., Fritzsche J., Langhammer C., Gómez Rivas J., Baldi A. (2022). Inverse designed plasmonic metasurface with parts per billion optical hydrogen detection. Nat. Commun..

[13] Tomeček D., Moberg H.K., Nilsson S., Theodoridis A., Darmadi I., Midtvedt D., Volpe G., Andersson O., Langhammer C. (2024). Neural network enabled nanoplasmonic hydrogen sensors with 100 ppm limit of detection in humid air. Nat. Commun..

[14] Girão A.F., Completo A. (2024). Eye-readable sensors for intuitive hydrogen monitoring. Int. J. Hydrogen Energy.

[15] Luo Y., Zhang C., Zheng B., Geng X., Debliquy M. (2017). Hydrogen sensors based on noble metal doped metal-oxide semiconductor: A review. Int. J. Hydrogen Energy.

[16] Li Q., Wang L., Xiao A., Zhu L., Yang Z. (2025). Hydrogen sensing towards palladium-based nanocomposites: A review. Int. J. Hydrogen Energy.

[17] Qureshi F., Yusuf M., Arham Khan M., Ibrahim H., Ekeoma B.C., Kamyab H., Rahman M.M., Nadda A.K., Chelliapan S. (2023). A State-of-The-Art Review on the Latest trends in Hydrogen production, storage, and transportation techniques. Fuel.

[18] Del Orbe Henriquez D., Cho I., Yang H., Choi J., Kang M., Chang K.S., Jeong C.B., Han S.W., Park I. (2021). Pt Nanostructures Fabricated by Local Hydrothermal Synthesis for Low-Power Catalytic-Combustion Hydrogen Sensors. ACS Appl. Nano Mater..

[19] Zhang W., Zou J., Sun J., Wang W., Shan L., Bao K., Gao W., Zhou Y., Jin Q., Jian J. (2024). Fabrication and sensing performance of carrier-free catalytic combustion hydrogen sensors based on electrodeposition method. Int. J. Hydrogen Energy.

[20] Panama G., Lee S.S. (2024). Catalytic thermoelectric hydrogen sensor with CoSb_3_ thermopile by single-step deposition on bare/textured glass. Surf. Interfaces.

[21] Seleka W.M., Phasha M.M., Kganyakgo L.K., Makwakwa D., Makhado E. (2025). Preparation of a conductive hydrogel based on carrageenan, polyvinyl alcohol, and polypyrrole as a potential room temperature electrochemical hydrogen gas sensor. Microchem. J..

[22] Lee E.G., Jung S.-W., Jo Y.E., Yoon H.R., Yoo B.K., Choi S.H., Choi J.W., Jang J.S., Lee S.-Y. (2022). Electrochemical Hydrogen Sensor Assembly for Monitoring High-Concentration Hydrogen. Phys. Status Solidi (a).

[23] Schwandt C. (2013). Solid state electrochemical hydrogen sensor for aluminium and aluminium alloy melts. Sens. Actuators B Chem..

[24] Alaghmandfard A., Fardindoost S., Frencken A.L., Hoorfar M. (2024). The next generation of hydrogen gas sensors based on transition metal dichalcogenide-metal oxide semiconductor hybrid structures. Ceram. Int..

[25] Mandal S., Marsh A.V., Faber H., Ghoshal T., Goswami D.K., Tsetseris L., Heeney M., Anthopoulos T.D. (2025). A robust organic hydrogen sensor for distributed monitoring applications. Nat. Electron..

[26] Zhong A., Sasaki T., Hane K. (2014). Platinum/porous GaN nanonetwork metal-semiconductor Schottky diode for room temperature hydrogen sensor. Sens. Actuators A Phys..

[27] Yang S., Dai J., Qin Y., Xiang F., Wang G., Yang M. (2018). Improved performance of fiber optic hydrogen sensor based on MoO_3_ by ion intercalation. Sens. Actuators B Chem..

[28] Zhang X., Zhang X., Li X., Liu Q., Zhang Y., Liang Y., Liu Y., Peng W. (2022). The nanophotonic machinal cavity and its hydrogen sensing application. Sens. Actuators B Chem..

[29] Wei X., Liang Y., Zhang X., Li R., Wei H., He Y., Shen L., Fang Y., Xu T., Peng W. (2025). Multi-resonance enhanced photothermal synergistic fiber-optic Tamm plasmon polariton tip for high-sensitivity and rapid hydrogen detection. Opto-Electron. Sci..

[30] Zhang X., Li X., Zhang X., Peng W. (2025). Fiber Optics-Mechanics Coupling Sensor for High-Performance Hydrogen Detection. Photonic Sens..

[31] Li Z., Xia X., Lu C., Yang Z., Yan X., You D., Li Z., Guo T. (2024). Ultrafast and Repeatable Optical Fiber Hydrogen Sensor with Urchin-Like Nanospheres Functionalization. IEEE Trans. Instrum. Meas..

[32] Khanikar T., Karki D., Su Y.D., Hong J.Y., Wang Y., Naeem K., Ohodnicki P.R. (2024). Pd/PMMA Nanocomposite-Coated Optical Fiber Hydrogen Sensor Operating at Room Temperature with Humidity Tolerance. IEEE Sens. J..

[33] Li J., Yan H., Dang H., Meng F. (2021). Structure design and application of hollow core microstructured optical fiber gas sensor: A review. Opt. Laser Technol..

[34] Li T., Zhao P., Wang P., Krishnaiah K.V., Jin W., Zhang A.P. (2024). Miniature optical fiber photoacoustic spectroscopy gas sensor based on a 3D micro-printed planar-spiral spring optomechanical resonator. Photoacoustics.

[35] Guo L., Bao H., Chen F., Zhao P., Jiang S., Ho H.L., Jin W. (2024). Ultra-Compact Optical Fiber Gas Sensor with High Sensitivity, Fast Response and Large Dynamic Range. J. Light. Technol..

[36] Zhang H., Bi S., Zhang Q., Tian C., Wang Z. (2023). The fiber ring laser intra-cavity gas sensor for C2H2 and CO2 detection based on photoacoustic spectroscopy. Infrared Phys. Technol..

[37] Zhang H., Bi S., Zhao J., Zhao Y., Qin L., Shi J., Fu Y., Wang Z. (2024). Dual-Gas Intra-Cavity QEPAS Sensor Based on Frequency Division Multiplexing Using Two Acoustic Microresonators. J. Light. Technol..

[38] Dang J., Zhang J., Chen T., Zheng C., Sun Y., Yu H., Chang Z. (2024). Simultaneous atmospheric CH_4_, CO_2_ and O_2_ detection using fiber-optic switch (FOS)-based time-division multiplexed NIR laser long optical path absorption spectroscopy. Sens. Actuators B Chem..

[39] Qin L., Bi S., Chen R., Zhao Y., Shi J., Zhang H., Wang Z. (2024). Two-Component Gas Sensor of Time-Division Multiplexing Technique Based on QEPAS and LITES. IEEE Photonics Technol. Lett..

[40] Peng M., Lu Z., Tang Y., Li C., Ren J., Dong C., Liu C. (2023). Femtosecond laser direct writing of long period fiber grating sensor with high refractive index sensitivity. Opt. Fiber Technol..

[41] Kang J., Liu C., Wei Y., Dang S., Wang X., Xiang H., Liu C., Shi C., Liu Z. (2025). High sensitivity and directional recognition torsion sensor based on rotating distributed slot long-period fiber grating. Measurement.

[42] Zhao Y., Hua Z., Hu M., Chen S., Mou C., Liu Y., He Z. (2024). High-Sensitivity Salinity Sensor Based on Helical Long-Period Fiber Grating in Thin-Cladding Fiber. J. Light. Technol..

[43] Chen Y., Yang Y., Liang C., Yao Y., Chen J. (2024). Palladium-based optical fiber Bragg grating hydrogen sensors: A comprehensive review. Opt. Laser Technol..

[44] Gao L., Tian Y., Hussain A., Guan Y., Xu G. (2024). Recent developments and challenges in resistance-based hydrogen gas sensors based on metal oxide semiconductors. Anal. Bioanal. Chem..

[45] Kumar V., Adalati R., Gautam Y.K., Gautam D. (2024). An investigation of glass, ITO, and quartz transparent substrates on Pd/SnO2 hydrogen sensor structure and sensitivity. Mater. Today Commun..

[46] Zhang H., Zhu H., Su H., Nie S., Zhu Y., Liu Y., Xu L. (2024). High performance potentiometric hydrogen sensor based on ZnO porous cage sensing electrode. Int. J. Hydrogen Energy.

[47] Chang C.-H., Chou T.-C., Chen W.-C., Niu J.-S., Lin K.-W., Cheng S.-Y., Tsai J.-H., Liu W.-C. (2020). Study of a WO_3_ thin film based hydrogen gas sensor decorated with platinum nanoparticles. Sens. Actuators B Chem..

[48] Nikolic M.V., Milovanovic V., Vasiljevic Z.Z., Stamenkovic Z. (2020). Semiconductor Gas Sensors: Materials, Technology, Design, and Application. Sensors.

[49] Hayashi Y., Yamazaki H., Ono D., Masunishi K., Ikehashi T. (2018). Investigation of PdCuSi metallic glass film for hysteresis-free and fast response capacitive MEMS hydrogen sensors. Int. J. Hydrogen Energy.

[50] Stephen W.J., Ralph P.T. (2003). Optical fibre long-period grating sensors: Characteristics and application. Meas. Sci. Technol..

[51] Esposito F., Srivastava A., Sansone L., Giordano M., Campopiano S., Iadicicco A. (2021). Label-Free Biosensors Based on Long Period Fiber Gratings: A Review. IEEE Sens. J..

[52] Sun C., Xu B., Li P. (2022). Hydrogen Sensor Based on NTC Thermistor with Pt-Loaded WO_3_/SiO_2_ Coating. Micromachines.

[53] Shen C., Huang Z., Chen X., Chen H., Wang Z., Li Y., Liu J., Zhou J. (2022). Reflective-Type High Sensitivity Optical Fiber Hydrogen Sensor Based On Enlarged Taper Cascaded with Tilted Fiber Grating. J. Light. Technol..

[54] Zhang X., Sun J., Tang K., Wang H., Chen T., Jiang K., Zhou T., Quan H., Guo R. (2022). Ultralow detection limit and ultrafast response/recovery of the H2 gas sensor based on Pd-doped rGO/ZnO-SnO_2_ from hydrothermal synthesis. Microsyst. Nanoeng..

