# Supplementary material for: Intrinsically Safe Optical Fiber Hydrogen Sensor Using Pt-SiO2 Coated Long-Period Fiber Grating
Source: Nanomaterials (Basel). 2026 Jan 12;16(2):95. doi: 10.3390/nano16020095 (PMC12844391; doi:10.3390/nano16020095)
Supplement: Supplementary file 1 [file nanomaterials-16-00095-s001.zip › nanomaterials-4075146-supplementary.pdf]

nanomaterials

Supplementary Materials

Intrinsically Safe Optical Fiber Hydrogen Sensor Using Pt-SiO_2 Coated Long-Period Fiber Grating

Xuhui Zhang [1], Liang Guo [1], Xinran Wei [2], Fangzhou Mao [1], Yuzhang Liang [2], Junsheng Wang [1,*] and Wei Peng [2]

[1] Liaoning Key Laboratory of Marine Sensing and Intelligent Detection, Information Science and Technology College, Dalian Maritime University, Dalian 116026, China
[2] School of Physics, Dalian University of Technology, Dalian 116024, China
* Correspondence: wangjsh@dlmu.edu.cn

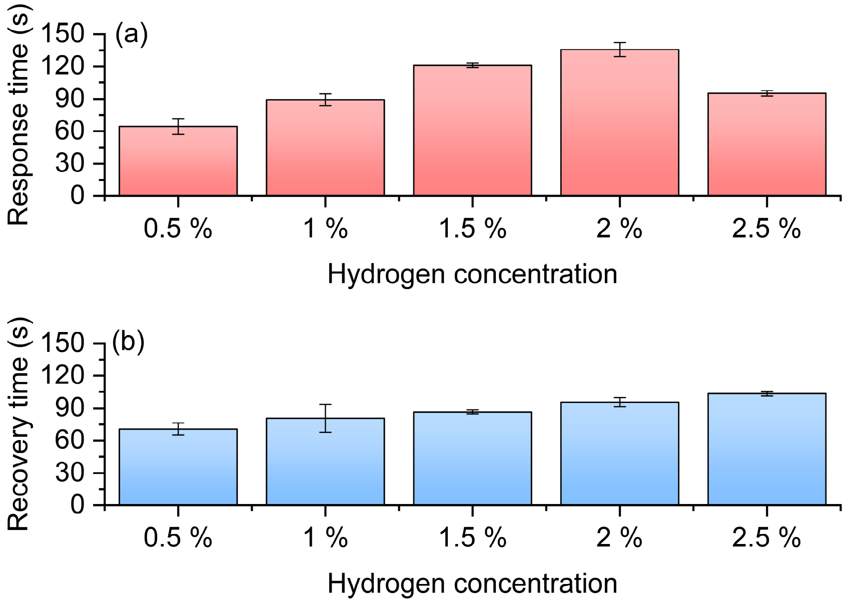

Figure S1. The repeatability of the response time and recovery time. (a) and (b) present the average response time and recovery time of the sensor at different hydrogen concentrations (0.5–2.5%).

Academic Editor: Marco Cannas

Received: 16 December 2025
Revised: 6 January 2026
Accepted: 9 January 2026
Published: 12 January 2026

Copyright: © 2026 by the authors. Submitted for possible open access publication under the terms and conditions of the Creative Commons Attribution (CC BY) license.

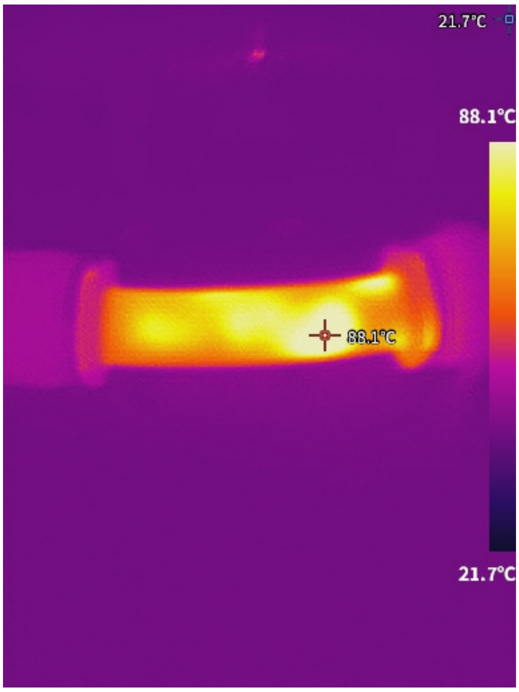

Figure S2. The infrared measurements.
